# New multiplex real-time PCR approach to detect gene mutations for spinal muscular atrophy

**DOI:** 10.1186/s12883-016-0651-y

**Published:** 2016-08-17

**Authors:** Zhidai Liu, Penghui Zhang, Xiaoyan He, Shan Liu, Shi Tang, Rong Zhang, Xinbin Wang, Junjie Tan, Bin Peng, Li Jiang, Siqi Hong, Lin Zou

**Affiliations:** 1 Center for Clinical Molecular Medicine, Children’s Hospital, Chongqing Medical University, 136 Zhongshan Er Road, Yuzhong District, Chongqing, 400014 China; 2 Center for Clinical Laboratory, Children’s Hospital, Chongqing Medical University, Yuzhong District, Chongqing, China; 3 Department of Neurology, Children’s Hospital, Chongqing Medical University, Yuzhong District, Chongqing, China; 4 Ministry of Education Key Laboratory of Development and Disorders, Children’s Hospital, Chongqing Medical University, Yuzhong District, Chongqing, China; 5 Key Laboratory of Pediatrics in Chongqing, Children’s Hospital, Chongqing Medical University, Chongqing, China; 6 Department of Health Statistics, School of Public Health, Chongqing Medical University, Yuzhong District, Chongqing, China

**Keywords:** Spinal muscular atrophy, Multiplex real-time PCR, Newborn screening

## Abstract

**Background:**

Spinal muscular atrophy (SMA) is the most common autosomal recessive disease in children, and the diagnosis is complicated and difficult, especially at early stage. Early diagnosis of SMA is able to improve the outcome of SMA patients. In our study, Real-time PCR was developed to measure the gene mutation or deletion of key genes for SMA and to further analyse genotype-phenotype correlation.

**Methods:**

The multiple real-time PCR for detecting the mutations of survival of motor neuron (*SMN*), apoptosis inhibitory protein (*NAIP*) and general transcription factor IIH, polypeptide 2 gene (*GTF2H2*) was established and confirmed by DNA sequencing and multiplex ligation-dependent probe amplification (MLPA). The diagnosis and prognosis of 141 hospitalized children, 100 normal children and further 2000 cases of dry blood spot (DBS) samples were analysed by this multiple real-time PCR.

**Results:**

The multiple real-time PCR was established and the accuracy of it to detect the mutations of *SMN*, *NAIP* and *GTF2H2* was at least 98.8 % comparing with DNA sequencing and MLPA. Among 141 limb movement disorders children, 75 cases were SMA. 71 cases of SMA (94.67 %) were with *SMN* c.840 mutation, 9 cases (12 %) with *NAIP* deletion and 3 cases (4 %) with *GTF2H2* deletion. The multiple real-time PCR was able to diagnose and predict the prognosis of SMA patients. Simultaneously, the real-time PCR was applied to detect trace DNA from DBS and able to make an early diagnosis of SMA.

**Conclusion:**

The clinical and molecular characteristics of SMA in Southwest of China were presented. Our work provides a novel way for detecting SMA in children by using real-time PCR and the potential usage in newborn screening for early diagnosis of SMA.

**Electronic supplementary material:**

The online version of this article (doi:10.1186/s12883-016-0651-y) contains supplementary material, which is available to authorized users.

## Background

Spinal muscular atrophy (SMA) is one of the most common autosomal recessive disorders, with an incidence of 1 in 10,000 births [1]. The disease is characterized by the degeneration of the anterior horn cells of the spinal cord, resulting in symmetrical limb muscle atrophy and weakness. SMA is classified into three clinical subtypes: Type I SMA (Werdnig-Hoffmann disease, MIM253300), Type II SMA (MIM253550) and Type III (Kugelberg-Welander disease, MIM253400) [2]. The phenotypes of these three subtypes of SMA are based on previous reports [3].

Previously, a clinical diagnosis of SMA is confirmed by muscle biopsy and electromyography (EMG). These procedures are time-consuming, affected by cross-talk and post-processing artefacts and yield non-conclusive results in young infants [4, 5]. Thus, the diagnosis of SMA is usually dependent on the doctor’s experience. When a diagnosis is made, these infants often suffer from irreversible loss of neurological function. Early diagnosis of SMA can improve outcomes, and the efficacy of early diagnosis of SMA is also motivated by progress in therapeutic development [6].

Due to the shortcomings of the traditional diagnosis of SMA, molecular diagnosis for SMA has gradually been developed in recent years. For the molecular genetics of SMA, all three clinical subtypes of SMA are associated with mutations in the survival of motor neuron (*SMN*) gene, which is mapped to chromosome 5q13. Normally, this region contains one telomeric *SMN*1 gene (Genbank: NG_008691.1) and one centromeric paralogue *SMN*2 gene (Genbank: NG_008728.1), which differs in exon 7 at cDNA residue 840 (C for *SMN*1 and T for *SMN*2) [7, 8]. Patients with a homozygous deletion of *SMN*1 and a high *SMN*2 copy number have a phenotype [8–10] due to the small fraction of normal transcripts, which indicates that infants with a homozygous mutation on c.840 of C > T will have symptoms of SMA. Moreover, the phenotype is correlated with the exon loss of several genes in the 5q13 region, such as apoptosis inhibitory protein (*NAIP*) (Genbank: NG_008724.1) and general transcription factor IIH, polypeptide 2 gene (*GTF2H2*) (Genbank: NT_187651.1) [11]. The absence of exon 4 and 5 of *NAIP* may result from an unequal crossing over, leading to severe SMA [12]. The *GTF2H2* gene and/or exon deletion might be related to the severity of the disease, but its clinical significance is still unclear [13].

The “gold standard” to detect a single nucleotide difference is DNA sequencing, and the best method to test for the exon loss of several genes is multiplex ligation-dependent probe amplification (MLPA) [11]. Only trace DNA can be extracted from a dry blood spot (DBS), which limits the application of DNA sequencing and MLPA during newborn screening, when is the best time to make an early diagnosis of SMA and treat in time, based on DBS being the only specimen could be obtained during newborn screening. Moreover, both of these methods are time-consuming. For restriction fragment length polymorphism (RFLP), which was established by Van’s group [14], it is also time-consuming, taking nearly 20 h to get the result, because the procedure contains PCR and enzyme digestion; on the other hand, it is hard to judge the results in daily work only by 22 base pairs differences through electrophoresis. Multiple real-time PCR has been used to detect single nucleotide differences and exon loss in adults and newborn screening [15, 16], which means multiple real-time PCR diagnosing SMA is very likely to be successful. Moreover, the technique is high sensitivity, specificity and rapid, so we chose it to establish a new method for SMA diagnosis. We screened and chose the best multiple pairs of primers and probes to establish the new real-time PCR method, which was fast (<3 h), cheap (<US$2), accurate, to analyse exons deletion of *SMN, NAIP* and *GTF2H2* genes.

In our study, the clinical and molecular characteristics of a pediatric population with SMA from Southwest China were presented. Real-time PCR was developed to measure the gene mutation or deletion of key genes for SMA and to further analyse genotype-phenotype correlation; subsequently, 2000 cases of DBS were randomly selected for trace DNA, and real-time PCR was performed.

## Methods

### Patients

This retrospective and prospective study was performed at the Children’s Hospital of Chongqing Medical University. In this study, 1613 children with limb movement disorders were screened, of which 141 children were confirmed to exhibit the inclusion criteria listed in the “Interpretation” section. These 141 children and an additional 100 normal children were enrolled in a retrospective study, and their blood specimens and medical records were collected. Simultaneously, 2000 DBS samples were randomly selected from the Newborn Screening Center at the Children’s Hospital of Chongqing Medical University to carry out the prospective study. This study was approved by the Ethics Committee of Children’s Hospital of Chongqing Medical University, and the reference number was CHCMU-20110005. Written informed consent was obtained from the parents or legal guardians of the patients. The datasets supporting the conclusions of this article are included within the article and its additional files.

### Interpretation

The inclusion criteria for the retrospective study were patients confirmed with a normal nutritional status; disorder on sitting, standing or walking; physical examination showing myasthenia or muscle atrophy; sequencing, multiplex PCR and MLPA detected disorder; and hospitalized. There was no inclusion criteria for the prospective study because the samples were selected randomly. The guidelines for SMA diagnosis and typing were based on a previous report [3] and were described in Additional file 1: Table S1.

### Real-time PCR for *SMN*, *NAIP* and *GTF2H2* genes in clinical specimens

All 141 patients and 100 normal children were enrolled. DNA was purified according to the manufacturer’s instructions (DP318-03, Tiangen Biotech, Beijing, China). Next, 1 μl of DNA template (20 ng/μl) and 19 μl of a real-time PCR master mix (FP203-02, Tiangen Biotech, Beijing, China), as shown in Additional file 2: Table S2, were used to perform real-time PCR. One sample should be detected by two real-time PCR reactions: tube I and tube II. Tube I detected the mutation of *SMN* gene and the deletion of *NAIP* exon5 and tube II detected internal control and the deletion of *NAIP* exon4 and *GTF2H2* exon 10.

The primers and probes used in this study are shown in Additional file 3: Table S3 and were synthesized by Invitrogen (Life Technologies Corporation, Shanghai, China). Real-time PCR was performed on the Applied Biosystem 7500 real-time PCR system (Applied Biosystems, CA, USA) using the following conditions: 50 °C for 2 min, 95 °C for 10 min, followed by 40 cycles of 95 °C for 15 s and 55 °C for 40 s. All standards and samples were performed in triplicate.

### Sequencing for *SMN* gene in clinical specimens

All 141 patients and 100 normal children were analysed using Sanger DNA sequencing for the point mutation at c.840 C > T of the *SMN* gene. The DNA template was the same template used in real-time PCR. Each PCR reaction was performed in a 50-μl volume, as shown in Additional file 4: Table S4. PCR was performed on an ABI-Verity Thermal Cycler (Applied Biosystems, Foster City, California, USA) using the following conditions: 95 °C for 5 min, followed by 35 cycles of 95 °C for 30 s, 53 °C for 30 s and 72 °C for 30 s, and a final extension step at 72 °C for 5 min. All samples were referred to Sunny (Sunny Biotechnology Co. Ltd., Shanghai, China) for sequencing.

### Multiplex PCR and MLPA for *NAIP* and *GTF2H2* genes in clinical specimens

All 141 patients and 100 normal children were analysed using multiplex PCR and MLPA for the *NAIP* and *GTF2H2* genes as previously reported [11, 17]. The DNA template was the same template that was used in real-time PCR. All of the conditions of each PCR reaction for multiplex PCR were the same as those used for sequencing. All of the samples were amplified in duplicate.

The PCR products of the *NAIP* and *GTF2H2* genes were used for DNA sequencing at Sunny (Sunny Biotechnology Co., Ltd., Shanghai, China) to confirm the expected products. If there were exons missing, as determined by electrophoresis, MLPA was performed on the DNA samples to confirm the results at Genesky (Genesky Biotechnologies Inc., Shanghai, China).

### Real-time PCR for *SMN*, *NAIP* and *GTF2H2* genes in DBS

Real-time PCR was performed on 2000 randomly selected DBS samples. DNA was purified by Chelex-100 (1422842, Bio-Rad Laboratories Co., Ltd., Shanghai, China). Briefly, one dried blood spot (diameter of 3 mm) was clipped and mixed with 500 μl of nuclease-free water to wash and was then centrifuged, and the supernatants were discarded. Next, 5 % Chelex-100 was mixed before the addition of 100 μl to each pellet, and the mixture was incubated at 56 °C for 10 min. The mixture was then mixed and centrifuged to harvest the supernatants.

Real-time PCR was performed on the DNA templates of the 6 μl and 14 μl PCR master mix, as shown in Additional file 5: Table S5. All of the real-time PCR conditions were the same as those described in the “Real-time PCR for *SMN*, *NAIP* and *GTF2H2* genes on clinical specimens” section.

### Statistics

The categorical data were expressed as percentages. Differences in the clinical characteristics of the various subtypes of SMA were determined using Fisher’s exact test. The survival rates of the different groups were analysed using log-rank. McNemar’s test was performed to analyse the paired results of diverse methods, and *p* < 0.05 was considered statistically significant. Data were analysed using software SAS 9.13 (SAS Institute, Cary, NC, USA).

## Results

### Patients and general characteristics

A total of 1613 children with limb movement disorders were initially investigated, and 141 child patients suspected to have SMA were enrolled in this study according to the inclusion criteria. The age of the children with limb movement disorders ranged from 1 month (m) to 15 years. The ratio of boys to girls was 1.660:1. The subtype of SMA was based on the onset age and clinical phenotypes [3], and the children in the study were divided into groups of ≤6 m, 6–18 m and ≥18 m. The general and clinical characteristics of the SMA-suspected children enrolled in the study are listed in Table 1.

**Table 1:** The general and clinical characteristics of children in the retrospective study

General and clinical characteristics	N (%)
All children	141 (100 %)
Gender	
Boy	88 (62.41)
Girl	53 (37.59)
Age	
< 6 m	60 (42.55)
7-18 m	45 (31.91)
> 18 m	36 (25.53)
Clinical characteristics	
Congenital heart disease	11 (7.80)
Respiratory failure	14 (9.93)
Muscular atrophy	38 (26.95)
EMG abnormalities	117 (82.98)
Disorder on sitting/standing/walking	64 (45.39)/39 (27.66)/29 (20.57)
Prenatal	
Ultrasonography abnormalities	3 (2.13)
Decreased fetal movement	6 (4.26)
Creatine kinase value	
Normal	50 (35.46)
Elevation	91 (64.54)
Diseases	
SMA	75
DMD/BMD	59
ME	6
CMT	1

There were 75 SMA children based on the SMA diagnosis guideline [3], and most of these children were grouped as Type I and Type II (Table 2). From this table, children with Type I SMA were more susceptible to congenital heart disease and respiratory failure and more prone to decreased fetal movement compared to children with Type II SMA. However, there were no significant differences between the two groups (Table 2). The remaining 66 children had Duchenne or Becker muscular dystrophy (DMD/BMD), mitochondrial encephalomyopathy (ME) and Charcot-Marie-Tooth (CMT) (Table 1).

**Table 2:** The general and clinical characteristics of children in different subtypes of SMA

	Type I (%) (*n* = 41)	Type II (%) (*n* = 29)	Type III (%) (*n* = 5)	Total (%) (*n* = 75)	*P* Value*
Gender*					
Boy	24 (58.54)	19 (65.52)	3 (60)	46 (61.33)	
Girl	17 (41.46)	10 (34.48)	2 (40)	29 (38.67)	
Clinical symptoms*					
Congenital heart disease	3 (7.32)	0	0	3 (4)	0.5137
Respiratory failure	6 (14.63)	2 (6.90)	0	8 (10.67)	0.7308
Muscular atrophy	20 (48.78)	13 (44.83)	2 (40)	35 (46.67)	0.9835
EMG abnormalities	39 (95.12)	28 (96.55)	4 (80)	71 (94.67)	0.4405
Prenatal*					
Ultrasonography abnormalities	0	0	0	0	-
Decreased fetal movement	3 (7.32)	2 (6.90)	0	5 (6.67)	1.0000
CK value*					
Normal	6 (14.63)	8 (27.59)	2 (40)	16 (21.33)	0.3391
Elevation	35 (85.37)	21 (72.41)	3 (60)	59 (78.67)	
Molecular detection					
Homozygous mutation at c.840 C > T in SMN gene	40 (97.56)	26 (89.66)	5 (100)	71 (94.67)	
Deletion of NAIP exon4 and 5	6 (14.63)	0	0	6 (8)	
Deletion of NAIP exon4	3 (7.32)	0	0	3 (4)	
Deletion of GTF2H2 exon10	2 (4.88)	1 (3.45)	0	3 (4)	

*Fisher’s exact test

### Molecular diagnosis and prognosis of SMA using novel real-time PCR methods

**Fig. 1:**
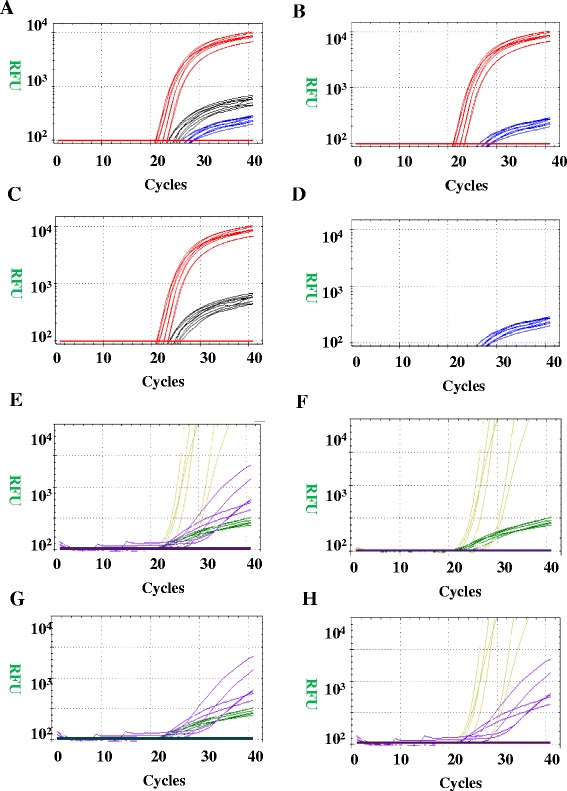
The amplification curves of real-time PCR. The threshold of RFU was set to be 10^2^. The red curve represented NAIP exon5; the black curve represented wild type of SMN gene; the blue curve represented mutation type of SMN; the yellow curve represented GTF2H2 exon10, the purple curve represented NAIP exon4 and the green curve represented internal control. **a** The image represented that the patient was only with heterozygous mutation of SMN and no mutation on NAIP exon5; (**b**) The image represented that the patient was with homozygous mutation of SMN and no mutation on NAIP exon5; (**c**) The image represented that the patient was normal for SMN and NAIP exon5; (**d**) The image represented that the patient was with homozygous mutation of SMN and the deletion of NAIP exon5; (**e**) The image represented that there was no mutation on GTF2H2 exon10 and NAIP exon4, and internal control was normal; (**f**) The image represented that the patient was with the deletion of NAIP exon4; (**g**) The image represented that the patient was with the deletion of GTF2H2 exon10; (**h**) The image represented that there was no amplification for internal control, and sample should be detected again

There were no significant differences of abnormal physical examination syndromes and laboratory detection between SMA and other neurological disorders, for instance, DMD/BMD [18]. The basic molecular diagnosis for SMA was performed by detecting the presence of a homozygous exon 7 deletion in the *SMN* gene, as previously reported [8, 9]. Deletions in the *NAIP* and *GTF2H2* genes were correlated with the severity and phenotype of SMA [12, 13], which contributed to SMA prognosis and typing. These real-time PCR results demonstrated a typical “S-shape” curve in different colors, and the threshold of relative fluorescent units (RFU) was set as 10^2^. The meaning of the colorful curves were shown in Fig. 1, and the real-time PCR results are shown in Fig. 1, indicating that this new real-time PCR was able to detect the exons with a homozygous loss of *SMN, NAIP* and *GTF2H2*.

There were 71 (94.67 %) SMA patients with homozygous deletion on *SMN*1 among 75 SMA patients; 6 SMA patients with a homozygous deletion of *NAIP* (exon 4 and exon 5), 3 SMA patients with a homozygous deletion of *NAIP* (exon 4) and 3 SMA patients with a homozygous deletion of *GTF2H2* (exon 10). Eleven of the patients were diagnosed as having Type I SMA, and the remaining patients were diagnosed as having Type II SMA (Table 2). The percentage of the *NAIP* and *GTF2H2* homozygous deletions were only 12 % (9/75) and 4 % (3/75), respectively, among all of the SMA children (Table 2).

### Consistency of *SMN*, *NAIP* and *GTF2H2* gene detection among Sanger DNA sequencing, MLPA and real-time PCR

**Fig. 2:**
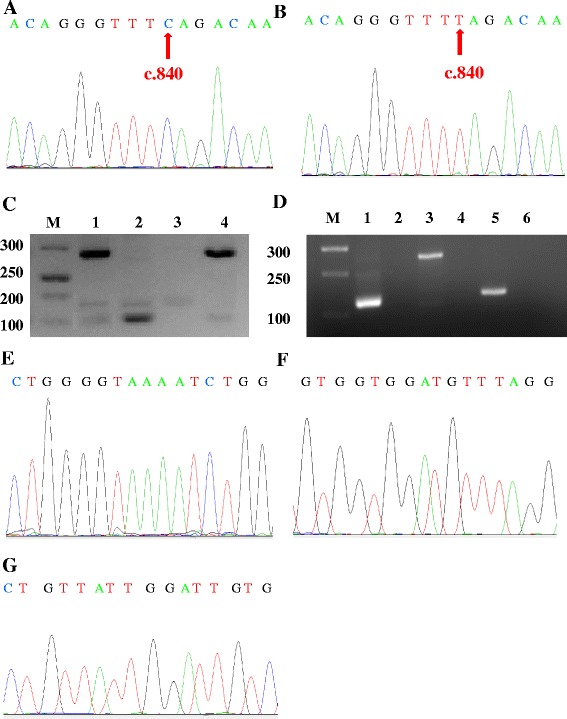
Detection SMN, NAIP and GTF2H2 genes by multiplex PCR and DNA Sanger sequencing. **a** The DNA sequence result of wild-type at c.840 of SMN (exon7); (**b**) The DNA sequence result of homozygous mutation of C > T at c.840 of SMN (exon7); (**c**) The multiplex PCR image for NAIP and GTF2H2 gene deletions. M, Marker; **a**, NAIP exon4; **b**, GTF2H2 exon10; **c**, NAIP exon5; (**d**): The single PCR image for wild type and NAIP or GTF2H2 gene deletions. M, Marker; 1, 3, 5, samples of wild type of NAIP and GTF2H2; 2, patients with NAIP exon5 deletion; 4, patients with NAIP exon4 deletion; 6, patients with GTF2H2 exon10 deletion; (**e**) The DNA sequence map of NAIP exon4; (**f**) The DNA sequence map of NAIP exon5; (**g**) The DNA sequence map of GTF2H2 exon10

The “gold standard” for detecting a single nucleotide difference and exon loss of several genes [11, 17] is DNA sequencing and MLPA, which were simultaneously used to confirm the results of our newly designed real-time PCR. Wild-type *SMN* and the homozygous mutation at c.840 C > T in the *SMN* gene were confirmed using Sanger DNA sequencing (Fig. 2a, b). The electrophoresis results of this new multiplex real-time PCR, including normal samples, samples with homozygous mutations in *NAIP* in exon 4, homozygous deletions of *NAIP* in exon4 and exon5 and homozygous deletions of *GTF2H2* in exon 10, were shown in Fig. 2c. The wild-type and patients with the deletion of *NAIP* (exon4 or 5) or *GTF2H2* (exon10) detected by single PCR were shown in Fig. 2d.

**Fig. 3:**
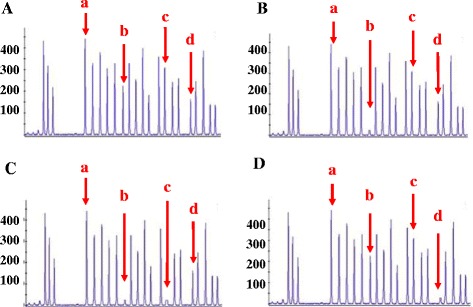
MLPA detection for NAIP and GTF2H2 genes. **a** The representative MLPA figures of normal sample; (**b**) The representative MLPA figures of NAIP exon4 deletion; (**c**) The representative MLPA figures of deletion of NAIP exon4 and 5; (**d**) The representative MLPA figures of GTF2H2 exon10 deletion. **a**: Internal control; **b**: Exon4 of NAIP; **c**: Exon5 of NAIP; **d**: Exon10 of GTF2H2

If there was no exon deletion in *SMN*, *NAIP* and *GTF2H2*, then all of the PCR products were confirmed using DNA Sanger sequencing (Fig. 2e, f and g). If any exon homozygous deletions were detected, then the abnormal and normal samples were confirmed using MLPA. There were four main peaks for normal samples, which indicated no deletion of the three exons of *NAIP* (exon 4 and exon 5) and *GTF2H2* (exon 10) (Fig. 3a). For the three different types of homozygous deletions of the *NAIP* or *GTF2H2* gene, one or more peaks were observed to be missing (Fig. 3b, c and d).

The results of this novel real-time PCR were compared with those obtained from DNA Sanger sequencing and multiplex PCR plus MLPA (Table 3). The accuracy of real-time PCR for the homozygous exon 7 deletion in the *SMN* gene was 99.6 % (240/241), and the false positive and negative rates of real-time PCR were 0 and 0.6 % (1/171), respectively, based on the results of DNA Sanger sequencing, indicating that this new real-time PCR was an ideal sequencing alternative for the detection of the *SMN* point mutation (Table 3). Simultaneously, the heterozygous *SMN* exon 7 deletion was found in no SMA patients, but was found in 62 children among the DMD/BMD, ME and normal patients, as confirmed using DNA Sanger sequencing (Table 4).

**Table 3:** The correlation of SMN, NAIP and GTF2H2 in different methods

	MLPA + multiplex PCR/Real-time PCR (%)				Sequencing/Real-time PCR (%)				*χ* ^2^	*P* value*
	+/+^a^	−/+ ^b^	+/− ^c^	−/− ^d^	+/+ ^e^	−/+ ^f^	+/− ^g^	−/− ^h^		
NAIP (exon4)	9 (3.7)	3 (1.2)	0	229 (95.1)					3.0	0.0833
NAIP (exon5)	6 (2.5)	2 (0.8)	0	233 (96.7)					2.0	0.1573
GTF2H2 (exon10)	3 (1.2)	2 (0.8)	0	236 (98.8)					2.0	0.1573
SMN					70 (29.1)	0	1 (0.4)	170 (70.5)	1.0	0.3173

^a^There were exons deletion of NAIP or GTF2H2 detected for both methods, meaning true positive for real-time PCR compared with MLPA + multiplex PCR

^b^There was no exon deletion of NAIP or GTF2H2 detected by MLPA + multiplex PCR but an exon deletion detected by real-time PCR, meaning false positive for real-time PCR compared with MLPA + multiplex PCR

^c^There was exon deletion of NAIP or GTF2H2 detected by MLPA + multiplex PCR but not detected by real-time PCR, meaning false negative for real-time PCR compared with MLPA + multiplex PCR

^d^There was no exon deletion for both MLPA + multiplex PCR and real-time PCR, meaning true negative for real-time PCR compared with MLPA + multiplex PCR

^e^There was homozygous mutation on SMN c.840 C > T for both methods, meaning true positive for real-time PCR compared with DNA sequencing

^f^There was no homozygous mutation on SMN c.840 C > T detected by Sanger DNA sequencing but detected by real-time PCR, meaning false positive for real-time PCR compared with DNA sequencing

^g^There was homozygous mutation on SMN c.840 C > T for Sanger DNA sequencing but not detected by real-time PCR, meaning false negative for real-time PCR compared with DNA sequencing

^h^There were normal and heterozygous mutation on SMN c.840 C > T for both Sanger DNA sequencing and real-time PCR, meaning true negative for real-time PCR compared with DNA sequencing

*McNemar test

**Table 4:** Heterozygous mutations of *SMN, NAIP* and *GTF2H2* genes among children

	SMN	NAIP		GTF2H2
	Exon7	Exon4	Exon5	Exon10
SMA	0	0	0	0
DMD/BMD	38	1	1	0
ME	1	0	0	0
CMT	0	0	0	0
Normal	23	7	3	4

Interestingly, the accuracy of real-time PCR for *NAIP* exon 4 and exon 5 was 98.8 % (238/241) and 99.2 % (239/241), respectively. The false positive rates of this new real-time PCR for *NAIP* exon 4 and 5 were 1.3 % (3/232) and 0.9 % (2/235), respectively, and the false negative rates of this new PCR for *NAIP* exon 4 and 5 were both 0. The accuracy of this novel real-time PCR for *GTF2H2* exon 10 was 99.2 % (239/241), and the false positive and negative rates were 0.8 % (2/238) and 0, respectively, based on the results of MLPA plus multiplex PCR in groups of SMA children and normal children. These results revealed that this new real-time PCR was an ideal substitution of MLPA for *NAIP* and *GTF2H2* mutation detection (Table 3). In addition, heterozygous deletions of *NAIP* or *GTF2H2* were found in 16 children, but no deletions were found in SMA patients using MLPA (Table 4).

### Prognosis of Type I SMA patients via the detection of *SMN*, *NAIP* and *GTF2H2* genes

**Fig. 4:**
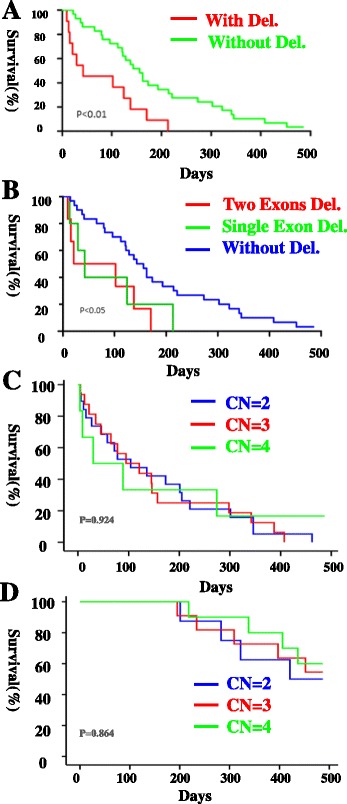
The survival analysis of type I SMA patients. **a** There were two groups of SMA type I patients: (1) patients with NAIP or GTF2H2 deletion, shown as red curve, *n* = 11; (2) patients without NAIP or GTF2H2 deletion, shown as green curve, *n* = 30. (**b**) SMA type I patients were classified into three groups: (1) patients with exon4 and 5 of NAIP deletion, shown as red curve, *n* = 6; (2) patients with one exon deletion in NAIP or GTF2H2, shown as green curve, *n* = 5; (3) patients without NAIP or GTF2H2 deletion, shown as blue curve, *n* = 30. (**c**) SMA type I patients were classified into three groups: (1) patients’ copy number (CN) of SMN2 = 2, shown as blue curve, *n* = 19; (2) patients’ copy number (CN) of SMN2 = 3, shown as red curve, *n* = 16; (3) patients’ copy number (CN) of SMN2 = 4, shown as green curve, *n* = 6. (**d**) SMA type II patients were classified into three groups: (1) patients’ copy number (CN) of SMN2 = 2, shown as blue curve, *n* = 8; (2) patients’ copy number (CN) of SMN2 = 3, shown as red curve, *n* = 11; (3) patients’ copy number (CN) of SMN2 = 4, shown as green curve, *n* = 10

The prognosis of patients is helpful to guide clinical treatment. Survival rate analysis was performed to evaluate the prognosis of patients with or without a homozygous gene deletion. There was only 1 patient with a homozygous deletion in *GTF2H2* diagnosed with Type II SMA, and thus, only the survival rate of Type I SMA patients was analysed. Among the 11 Type I SMA children, there was no patient with a complex gene deletion of *NAIP* or *GTF2H2*. The survival rates between Type I SMA patients with a homozygous deletion of *NAIP* or *GTF2H2* and patients without a deletion in *NAIP* or *GTF2H2* were analysed (Fig. 4a). There was a much lower survival rate in patients with a homozygous deletion in *NAIP* or *GTF2H2* (*p* < 0.01), particularly in the first 2 months after suffering from pneumonia (Fig. 4a).

Due to the different types of gene deletions, we determined if there was a difference in the prognosis among patients with a homozygous deletion in one exon, two exons, or no exons in SMA children. Type I SMA patients were divided into three groups: patients without a deletion in *NAIP* or *GTF2H2*, patients with a homozygous deletion in one exon of *NAIP* or *GTF2H2*, and patients with a homozygous deletion in exon 4 and 5 of *NAIP* (Fig. 4b). There was a much lower survival rate of patients with a homozygous deletion in one or two exons compared with patients without a deletion (*p* < 0.05), but there was no significant difference between patients with a homozygous deletion in one exon and those with a homozygous deletion in exon 4 and 5 of *NAIP* in Type I SMA patients.

According to He’s report [11], the copy number of *SMN2* was related to prognosis. In our study the copy number of *SMN2* varied from 2–4 of our SMA patients, as shown in Table 5. The relationship between the copy number of *SMN2* and patients’ prognosis was analysed, as shown in Fig. 4c, d. There were no significant differences among patients with different copy number of *SMN2* in Type I and Type II SMA patients (*p* > 0.05) in our study. All the patients with Type III SMA were still alive.

**Table 5:** *SMN2* copy number in different sub-types of SMA

SMA sub-types	*SMN2* copy number			Total
	2	3	4	
Type I	19 (46.34)	16 (39.02)	6 (14.63)	41
Type II	8 (27.59)	11 (37.93)	10 (34.48)	29
Type III	1 (20)	2 (40)	2 (40)	5
Total	28 (37.33)	29 (38.67)	18 (24)	75

### Novel real-time PCR for trace DNA

Although real-time PCR can replace DNA Sanger sequencing and MLPA for detecting mutations of *SMN*, *NAIP* and *GTF2H2*, it is necessary to determine whether it can function in newborn screening with trace DNA samples. We randomly selected 2000 DBS samples from 2011 to 2014 using the new real-time PCR method for the detection gene mutations (Table 6).

**Table 6:** Real-time PCR for Newborn screening

	2011	2012	2013	2014 (up to July)
DBS screened/Total	213/33499	637/99841	706/110592	444/69224
Positive number	0	0	1	0

All trace DNA samples were successfully collected and purified from the 2000 DBS samples. There were 23 samples detected as positive in the first run and all of them were reconfirmed by our Real-time PCR and then DNA sequenced. Twenty-two of them were proven to be false-positive, so there was only one sample, whose ID number was NS-13050012. This patient was first hospitalized due to pneumonia and myasthenia when he was 1 month old in May 2013, who was homozygous for the *SMN* exon 7 deletion, and the remaining 1999 samples were negative for *SMN*, *NAIP* and *GTF2H2* deletion. For the negative samples, 1997 of the patients had no symptoms related to SMA and 2 of the patients had limb movement disorders after a follow-up investigation. The 2 children that had limb movement disorders were subsequently diagnosed with progressive muscular dystrophy.

On the other hand, we collected peripheral blood from new SMA patients to make DBS from January 1^st^ to May 16^th^. There were 36 SMA patients. The results were shown in Table 7. It was obvious that the ability to detect DNA from DBS of our novel real-time PCR was the same as detecting DNA from peripheral blood. The low positive rate of the method in newborn screening DBS was related to the samples, which only contained 1 SMA patient, not because of the limit of our real-time PCR.

**Table 7:** Detection of SMA through DNA from peripheral blood or from DBS

	SMN deletion	NAIP deletion		GTF2H2 deletion
	Exon7	Exon4	Exon5	Exon10
Peripheral blood (*n* = 36)	35	4	1	0
DBS (*n* = 36)	35	4	1	0

## Discussion

Here, we established a novel real-time PCR method for detecting mutations in the *SMN*, *NAIP* and *GTF2H2* genes. The accuracy of this real-time PCR method for detecting the presence of a *SMN* point mutation or a homozygous deletion in *NAIP*, *GTF2H2* exons was at least 98.8 % compared with DNA Sanger sequencing and MLPA. Real-time PCR is a fast and an ideal method to replace DNA Sanger sequencing, multiplex PCR, and MLPA for the diagnosis of SMA and requires small amounts of DNA [19]. Real-time PCR is also suitable for application in newborn screening for SMA, similar to Somech’s report of T cell receptor excision circles in combined T and B cell immunodeficiency [20]. However, real-time PCR was not able to distinguish between different sub-types of SMA patients. This may be due to the correlation between the genotypes and phenotypes of SMA with hundreds of genes that are not located on chromosome 5q [21, 22], and thus, whole genome sequencing should be further explored.

The incidence of SMA was 4.65 % (75/1613) in children with limb movement disorders in the southwest part of China. Interestingly, most of our SMA patients were classified as Type I and II (Table 1). Type I SMA children were more susceptible to congenital heart diseases and respiratory failure and were more prone to decreased fetal movement compared with Type II SMA children, consistent with previous reports [23–25]. Neither the proportion of muscular atrophy abnormalities nor EMG were found in different subtypes of SMA patients.

In our SMA children, 94.67 % (71/75) displayed a homozygous *SMN*1 exon 7 deletion, which was similar to the findings obtained in a previous report [26]. However, the odds ratios of *NAIP* and *GTF2H2* homozygous deletions were only 12 % and 4 %, respectively, among SMA patients, which was much lower than the report in east China [11], indicating the different inheritance characteristics for SMA in southwest China. In our study, there was no heterozygous mutations or deletions of *SMN*, *NAIP* and *GTF2H2* found in SMA patients. Indeed, the multiplex real-time PCR detected a *SMN* heterozygous exon 7 deletion, as shown in Fig. 1a. In addition, real-time PCR detected heterozygous deletions of the *NAIP* or *GTF2H2* gene when a normal sample with no deletion of the genes was used as an internal control and an equal amount of DNA from patient was amplified (data not shown). Not only patients with DMD/BMD or ME and normal children, as shown in Table 4, but also 20 pairs of the parents of SMA patients (data not shown), who had no symptom, were found to have heterozygous mutations of *SMN1*, *NAIP* and *GTF2H2*. Some of them had no clinical symptom and some of them had clinical symptoms not related to mutations of *SMN1*, *NAIP* and *GTF2H2.* There was no clinical significance for heterozygous mutations of *SMN1*, *NAIP* and *GTF2H2.*

Among the Type I SMA patients, the development of SMA was much faster in patients with a homozygous deletion in *NAIP* or *GTF2H2* compared to patients without this type of deletion. For the 11 Type I SMA patients, 3 patients demonstrated decreased fetal movement during pregnancy. Among these 3 patients, the patient with a homozygous deletion in exon 4 of *NAIP* had a poor prognosis (the child died within the first 2 months). The amount of samples was too small to obtain a conclusion that decreased fetal movement may signal a bad prognosis for SMA patients. Thus, more cases of SMA children should be further investigated.

The relationship between the prognosis of patients and SMN2 copy number in our study was different from others [11, 27]. There were no significant differences between the possibility of developing to different sub-types of SMA and *SMN2* copy numbers in our study according to Table 5, whereas an inverse relationship between *SMN2* copy number and possibility of developing to Type I SMA in He J’s research. Maybe different genetic background result in this different prognosis comparing with He J’s research. The onset age of most of our type I SMA patients (38/41, 92.7 %) was <2 months and 72.4 % (21/29) of type II SMA patients carried more than 3 copies of *SMN2*, whereas the onset age of Qu’s type I patients was 31.1 % (33/106) and copies of *SMN2* of Qu’s type II patients was 96 %. As Qu’s research, the clinical and genetic characters of our patients were more severe, leading to worse survival rate.

Only one DBS-positive sample, whose ID number was NS-13050012, was analysed in our study. The doctor diagnosed that the baby suffered from Type I SMA using DNA sequencing and MLPA. The child was nursed as a Type I SMA patient and was still alive until September 2014, with no obvious symptoms except non-sitting. If we performed the newborn screening for SMA for newborn babies, children with SMA will benefit from being diagnosed as Type I SMA at an early age and can begin an early specific nursing program. Thus, their prognosis will improved, similar to the child in our study.

There are four main methods to detect SMA: Sanger DNA sequencing, MLPA, RFLP and multiple routine PCR. No one was used in newborn screening. Sanger DNA sequencing and MLPA are both time consuming (>2 weeks), expensive (>US$21) and high amount of DNA required (amount of DNA is trace in newborn screening); RFLP is also time consuming (20 h) and not able to detect exons deletion of *NAIP* and *GTF2H2*; multiplex routine PCR is not able to detect exon 7 deletion of *SMN1* and it is more subjective because of being judged by electrophoresis gel image; even the method of RFLP with multiplex routine PCR is also time consuming and subjective to judge results. Our new approach is fast (<3 h), cheap (<US$2), objective and able to analyse exons deletion of *SMN, NAIP* and *GTF2H2* genes simultaneously with trace DNA.

According to Prior’s and Stabley’s studies [28, 29], there are several limitations of real-time PCR. At the same time, digital PCR (dPCR) is able to detect SMN mutation. Moreover, for the coefficients of variation, dPCR is even better than real-time PCR.

For limitations of real-time PCR, (1) we recommended eathylene diamine tetraacetic acid (EDTA) as anticoagulant for periphery blood sample, avoiding heparin for its inhibiting Taq polymerase activity. Our method detected more than 200 EDTA peripheral blood and 2000 DBS samples, and the amplification efficiency was excellent, so the compounds in specimen did not shown inhibition; (2) it is possible that DNA sequence variants located under the primer binding sites may be a problem to influence the results. To avoid the question, we need to add another pair of primers to cover outside of binding sites of the primers we used to amplify samples in future; (3) for data analysis, repeating reactions twice and evaluating the capability of equipment could ensure the quality of results to a certain extent.

The recent new method of dPCR is suitable and promising for detecting SMN mutation. However, the ability of dPCR to simultaneously detect NAIP and GTF2H2 mutation and for DBS is not available and need further evaluation. Moreover, dPCR is more expensive (>US$15) in China and specific equipment is required. In a word, the Real-time PCR for SMN, NAIP and GTF2H2 is cheaper and feasibility for clinical usage at present in China.

## Conclusions

In summary, we have provided a better understanding of the incidence, clinical and laboratory characteristics of SMA in southwest China and established a new fast, cheap and accurate real-time PCR method for detecting SMA during newborn screening, which will be helpful in maintaining the motor function of Type I and II SMA patients in the near future (ClinicalTrial.gov. Safety and efficacy of olesoxime (TRO19622) in 3–25 years SMA patients. From http://clinicaltrials.gov/show/NCT01302600. Accessed October 8, 2013). Combined with SMA therapy development, SMA patients may also be specifically treated in a more timely fashion in the future [6]. Furthermore, whole genome sequencing should be explored to determine the genotype-phenotype correlation and typing of SMA.

## Abbreviations

DBS, dry blood spot; dPCR, digital PCR; EDTA, eathylene diamine tetraacetic acidEMG, electromyography; GTF2H2, general transcription factor IIH, polypeptide 2 gene; MLPA, multiplex ligation-dependent probe amplification; NAIP, NLR family, apoptosis inhibitory protein; RFLP, restriction fragment length polymorphism; RFU, relative fluorescent units; SMA, spinal muscular atrophy; SMN, survival of motor neuron

## Additional files

Additional file 1: Table S1. The guideline for SMA diagnosis and typing. (DOC 34 kb) Additional file 2: Table S2. The components of each real-time PCR reaction. (DOC 36 kb) Additional file 3: Table S3. The sequence of primers and probes. (DOC 37 kb) Additional file 4: Table S4. The reaction systems for DNA sequencing and multiplex PCR. (DOC 38 kb) Additional file 5: Table S5. The master mix of real-time PCR for DBS. (DOC 36 kb)
