# Supplementary material for: New multiplex real-time PCR approach to detect gene mutations for spinal muscular atrophy
Source: BMC Neurol. 2016 Aug 17;16:141. doi: 10.1186/s12883-016-0651-y (PMC4989483; doi:10.1186/s12883-016-0651-y)
Supplement: Additional file 1: Table S1. — The guideline for SMA diagnosis and typing. (DOC 34 kb) [file 12883_2016_651_MOESM1_ESM.doc]

**Suppl. Table 1 The guideline for SMA diagnosis and typing**

| SMA Type | Age of Onset | Age of Death | Typical Features |
| --- | --- | --- | --- |
| Type I | <6 months | <2 years | 1.Never sit  2.Profound weakness and  hypotonia,  3.Impaired head control  4.Difficulty with swallowing and handling of oral secretion  5. Early morbidity due to respiratory insufficiency and aspiration pneumonia |
| Type II | 7-18 months | >2 years | 1.Never stand  2. Delayed motor milestones  3.Weak cough  4.Joint contractures and  scoliosis  5.Death due to respiratory infection or failure |
| Type III | >18 months | Adult | 1.Stand and walk  2.Walk abnormally  3.Variable muscle weakness  and cramp  4.joint overuse, |
