# Supplementary material for: New multiplex real-time PCR approach to detect gene mutations for spinal muscular atrophy
Source: BMC Neurol. 2016 Aug 17;16:141. doi: 10.1186/s12883-016-0651-y (PMC4989483; doi:10.1186/s12883-016-0651-y)
Supplement: Additional file 3: Table S3. — The sequence of primers and probes. (DOC 37 kb) [file 12883_2016_651_MOESM3_ESM.doc]

**Suppl. Table 3 The sequence of primers and probes**

| Target | Primer Sequence | Primers Applications | Probe Sequence |
| --- | --- | --- | --- |
| SMN  (c.840 C>T) | Forward (SMN-7F):  5’-ACTTCCTTTATTTTCCTT-3’  Reverse (SMN-7R):  5’-ATGCTGGCAGACTTACTC-3’ | Multiplex PCR  Sequencing  Real-time PCR | Wild (SMN-W):  HEX-5’-AGGGTTTCAGACAAA-3’-TAMRA  Mutation (SMN-M):  FAM-5’-AGGGTTTtAGACAAA-3’-TAMRA |
| NAIP  (exon4) | Forward (NA-4F):  5’-CACAATTTGCTGCCAGAG-3’  Reverse (NA-4R):  5’-CGGCACCAAAGAGGATTA-3’ | Multiplex PCR  Sequencing  Real-time PCR | NA-4P  HEX-5'-TACAGCAGAAGCACT-3'-TAMRA |
| NAIP  (exon5) | Forward (NA-5F):  5’-CACTGCCAGGCAATCTAA-3’  Reverse (NA-5R):  5’-CATCTCCTTCTTCCCAAT-3’ | Multiplex PCR  Sequencing  Real-time PCR | NA-5P  Cy5-5’-TCCTAAACATCCACC-3’-BHQ3 |
| GTF2H2 (exon10) | Forward (GT-10F):  5’-TTCTTCTTTCCTAAAACTAT-3’  Reverse (GT-10R):  5’-ACCAGTTTCACGAGCAAG-3’ | Multiplex PCR  Sequencing  Real-time PCR | GT-10P  Cy5-5’-ACAGTGCAAACGCGA-3’-BHQ3 |
| GAPDH | Forward (GA-F):  5’-CATGAGAAGTATGACAACAGCCT -3’  Reverse (GA-R):  5’-AGTCCTTCCACGATACCAAAGT -3’ | Real-time PCR | GA-P  FAM-5’-ATCATCAGCAATGCCTCCT-3’-TAMRA |
