# Supplementary material for: New multiplex real-time PCR approach to detect gene mutations for spinal muscular atrophy
Source: BMC Neurol. 2016 Aug 17;16:141. doi: 10.1186/s12883-016-0651-y (PMC4989483; doi:10.1186/s12883-016-0651-y)
Supplement: Additional file 4: Table S4. — The reaction systems for DNA sequencing and multiplex PCR. (DOC 38 kb) [file 12883_2016_651_MOESM4_ESM.doc]

**Suppl. Table 4 The reaction systems for real-time PCR, DNA sequencing and multiplex PCR**

| DNA sequencing | | Multiplex PCR | |
| --- | --- | --- | --- |
| Components | Volume (μl) | Components | Volume (μl) |
| 2×Master Mix | 25 | 2×Master Mix | 25 |
| Forward primer (20 μmol/L) | 1 | DNA template (30ng/ul) | 4 |
| Reverse primer (20 μmol/L) | 1 | Nuclease-free water | 15 |
| DNA template (30ng/ul) | 4 | NA-4F (20umol/L) | 1 |
| Nuclease-free water | 19 | NA-4R (20umol/L) | 1 |
|  |  | GT-10F (20umol/L) | 1 |
|  |  | GT-10R (20umol/L) | 1 |
|  |  | GA-F (20umol/L) | 1 |
|  |  | GA-R (20umol/L) | 1 |
| Total | 50 | Total | 50 |
