# Supplementary material for: New multiplex real-time PCR approach to detect gene mutations for spinal muscular atrophy
Source: BMC Neurol. 2016 Aug 17;16:141. doi: 10.1186/s12883-016-0651-y (PMC4989483; doi:10.1186/s12883-016-0651-y)
Supplement: Additional file 5: Table S5. — The master mix of real-time PCR for DBS. (DOC 36 kb) [file 12883_2016_651_MOESM5_ESM.doc]

**Suppl. Table 5 The master mix of real-time PCR for DBS**

| Reaction I Tube | | Reaction II Tube | |
| --- | --- | --- | --- |
| Components | Volume (μl) | Components | Volume (μl) |
| 2.5×Real-MasterMix | 8 | 2.5×Real-MasterMix | 8 |
| 20×Probe Enhancer | 1 | 20×Probe Enhancer | 1 |
| SMN-7F (20umol/L) | 0.5 | NA-4F (20umol/L) | 0.5 |
| SMN-7R (20umol/L) | 0.5 | NA-4R (20umol/L) | 0.5 |
| SMN-W (20umol/L) | 0.5 | NA-4P (20umol/L) | 0.5 |
| SMN-M (20umol/L) | 0.5 | GT-10F (20umol/L) | 0.5 |
| NA-5F (20umol/L) | 0.5 | GT-10R (20umol/L) | 0.5 |
| NA-5R (20umol/L) | 0.5 | GT-10P (20umol/L) | 0.5 |
| NA-5P (20umol/L) | 0.5 | GA-F (20umol/L) | 0.5 |
| Nuclease-free Water | 1.5 | GA-R (20umol/L) | 0.5 |
|  |  | GA-P (20umol/L) | 0.5 |
|  |  | Nuclease-free Water | 0.5 |
| Total | 14 | Total | 14 |
